# A phase I/II study of the safety and efficacy of [^177^Lu]Lu-satoreotide tetraxetan in advanced somatostatin receptor-positive neuroendocrine tumours

**DOI:** 10.1007/s00259-023-06383-1

**Published:** 2023-09-18

**Authors:** Damian Wild, Henning Grønbæk, Shaunak Navalkissoor, Alexander Haug, Guillaume P. Nicolas, Ben Pais, Catherine Ansquer, Jean-Mathieu Beauregard, Alexander McEwan, Michael Lassmann, Daniele Pennestri, Magali Volteau, Nat P. Lenzo, Rodney J. Hicks

**Affiliations:** 1 Division of Nuclear Medicine, ENETS Centre of Excellence, University Hospital Basel, Basel, Switzerland; 2 Department of Hepatology & Gastroenterology, ENETS Centre of Excellence, Aarhus University Hospital and Clinical Institute, Aarhus University, Aarhus, Denmark; 3 Neuroendocrine Tumour Unit, ENETS Centre of Excellence, Royal Free London NHS Foundation Trust, London, UK; 4 Department of Radiology and Nuclear Medicine, Medical University of Vienna, Vienna, Austria; 5 SRT-Biomedical B.V., Soest, Netherlands; 6 Ariceum Therapeutics GmbH, Berlin, Germany; 7 CHU Nantes, Nantes Université, Médecine Nucléaire, Nantes, France; 8 Department of Medical Imaging, CHU de Québec – Université Laval, Quebec City, Canada; 9 Department of Nuclear Medicine, University Hospital Würzburg, Würzburg, Germany; 10 Ipsen, Slough, UK; 11 Ipsen, Les Ulis, France; 12 GenesisCare, East Fremantle, Australia; 13 Department of Medicine, Curtin University, Perth, Australia; 14 Department of Medicine, St Vincent’s Hospital, The University of Melbourne, Melbourne, Australia; 15 Department of Medicine, Central Clinical School, The Alfred Hospital, Monash University, Melbourne, Australia

**Keywords:** [^177^Lu]Lu-satoreotide tetraxetan, Clinical trial, Somatostatin receptor antagonist, Neuroendocrine tumours, Peptide receptor radionuclide therapy

## Abstract

**Purpose:**

We present the results of an open-label, phase I/II study evaluating the safety and efficacy of the novel somatostatin receptor (SSTR) antagonist [^177^Lu]Lu-satoreotide tetraxetan in 40 patients with previously treated, progressive neuroendocrine tumours (NETs), in which dosimetry was used to guide maximum administered activity.

**Methods:**

This study was conducted in two parts. Part A consisted of 15 patients who completed three cycles of [^177^Lu]Lu-satoreotide tetraxetan at a fixed administered activity and peptide amount per cycle (4.5 GBq/300 µg). Part B, which included 25 patients who received one to five cycles of [^177^Lu]Lu-satoreotide tetraxetan, evaluated different administered activities (4.5 or 6.0 GBq/cycle) and peptide amounts (300, 700, or 1300 μg/cycle), limited to a cumulative absorbed radiation dose of 23 Gy to the kidneys and 1.5 Gy to the bone marrow.

**Results:**

Median cumulative administered activity of [^177^Lu]Lu-satoreotide tetraxetan was 13.0 GBq over three cycles (13.1 GBq in part A and 12.9 GBq in part B). Overall, 17 (42.5%) patients experienced grade ≥ 3 treatment‑related adverse events; the most common were lymphopenia, thrombocytopenia, and neutropenia. No grade 3/4 nephrotoxicity was observed. Two patients developed myeloid neoplasms considered treatment related by the investigator. Disease control rate for part A and part B was 94.7% (95% confidence interval [CI]: 82.3–99.4), and overall response rate was 21.1% (95% CI: 9.6–37.3).

**Conclusion:**

[^177^Lu]Lu-satoreotide tetraxetan, administered at a median cumulative activity of 13.0 GBq over three cycles, has an acceptable safety profile with a promising clinical response in patients with progressive, SSTR-positive NETs. A 5-year long-term follow-up study is ongoing.

**Trial registration:**

ClinicalTrials.gov, NCT02592707. Registered October 30, 2015.

**Supplementary Information:**

The online version contains supplementary material available at 10.1007/s00259-023-06383-1.

## Introduction

Peptide receptor radionuclide therapy (PRRT) with radiolabelled somatostatin receptor (SSTR) agonists has shown to be well tolerated and effective, thereby becoming integral in management of advanced neuroendocrine tumours (NETs) [1–3]. Radiolabelled SSTR antagonists may further improve responses, with data demonstrating higher uptake and longer retention in tumours compared with SSTR agonists, despite lack of internalisation of the ligand-receptor complex [2, 4, 5]. Their higher tumour uptake appears to reflect affinity for a greater number of receptor binding sites and slower dissociation, allowing for a longer accumulation of, and cellular exposure to, ionising beta radiation [2, 6, 7].

[^177^Lu]Lu-satoreotide tetraxetan (also known as [^177^Lu]Lu-SSO110, [^177^Lu]Lu-IPN01072, [^177^Lu]Lu-OPS201, or [^177^Lu]Lu-DOTA-JR11) is a novel SSTR antagonist, which showed higher and more stable tumour uptake in pre-clinical models and more pronounced cytotoxic treatment effect in comparison to the SSTR agonists [^177^Lu]Lu-DOTA-TATE and [^177^Lu]Lu-DOTATOC [8, 9]. In a comparison of [^177^Lu]Lu-satoreotide tetraxetan and [^177^Lu]Lu-DOTA-TATE in four patients with progressive NETs, [^177^Lu]Lu-satoreotide tetraxetan was associated with a 1.3–2.8-times longer intra-tumoural residence time and 1.1–2.6-times higher tumour uptake, resulting in a 1.7–10.6-times higher tumour absorbed dose with an acceptable toxicity profile [10]. A phase I study involving 20 patients with pre-treated NETs reported a median progression-free survival (PFS) of 21.0 months and a disease control rate (DCR) of 85.0% after [^177^Lu]Lu-satoreotide tetraxetan therapy [11]; in this study, no renal toxicity was observed, and an acceptable haematologic toxicity profile was achieved following reduction of the administered activity of [^177^Lu]Lu-satoreotide tetraxetan [11].

We present the results of a phase I/II study evaluating the safety and efficacy of [^177^Lu]Lu-satoreotide tetraxetan in patients with progressive, SSTR-positive NETs. [^177^Lu]Lu‑satoreotide tetraxetan treatment was adapted to limit the absorbed radiation doses to pre-specified thresholds for the bone marrow and kidneys (the dose-limiting organs for PRRT) [12]. A plain language summary of this publication can be found in the Supplementary Information.

## Materials and methods

### Study design

A phase I/II, multinational, multicentre, open-label study (NCT02592707) was conducted in eight centres across Australia, Europe, and Canada, in accordance with the Declaration of Helsinki and the International Conference on Harmonisation Good Clinical Practice guidelines. All patients provided written informed consent. Ethical approval was obtained from relevant ethics committees. Patient enrolment was initiated on 6 March 2017, with data cut-off for this analysis on 1 April 2021.

**Fig. 1:**
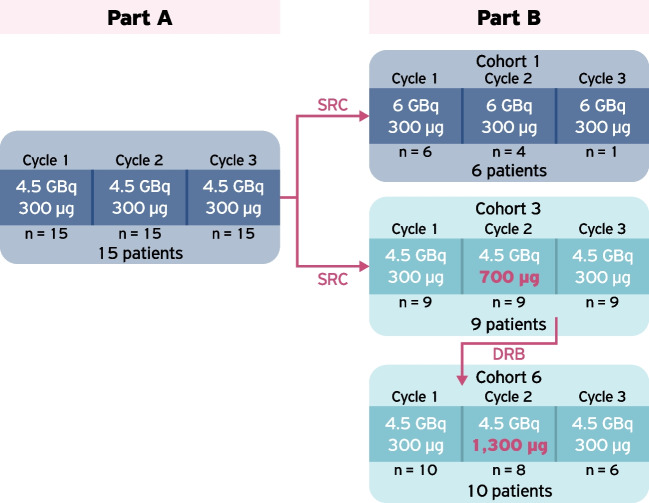
Administered activity and peptide amounts of [^177^Lu]Lu-satoreotide tetraxetan in cycles 1 to 3 of part A and part B of the study. Note that four patients in cohort 3 and two patients in cohort 6 received additional treatment cycles (see Table 2). The study had a SRC (part A) and a DRB (part B). During part B, each escalation cohort (whether of administered activity or peptide amount) was evaluated by the DRB. *DRB* Data Review Board, *SRC* Safety Review Committee

The study was conducted in two parts for safety reasons (Fig. 1). Part A consisted of 15 patients administered with three cycles of [^177^Lu]Lu-satoreotide tetraxetan at an activity of 4.5 GBq (± 10%) and a total peptide amount of 300 µg (± 50). The intended cumulative administered activity for part A was 13.5 GBq. Initially, six patients were treated with three cycles of [^177^Lu]Lu-satoreotide tetraxetan; following a Safety Review Committee (SRC) meeting, it was decided that the remaining nine patients in part A could be exposed to the planned administration.

Subsequently, after evaluation of safety and dosimetry data from part A by the SRC, part B was initiated. Patient safety and escalation of administered activity were evaluated by a Data Review Board (DRB) in part B. Part B assessed different administered activities and peptide amounts of [^177^Lu]Lu-satoreotide tetraxetan, as both the effect of increasing peptide amount, and the peptide amount at which lesion receptor saturation occurs in humans, are unknown. A previous study in a xenograft mouse model demonstrated that increasing peptide amounts resulted in a reduced absorbed dose in non-target organs without compromising tumour uptake, and no saturation of the receptor was observed with higher peptide amounts [13].

Part B consisted of 25 patients in three cohorts (cohort 1, *N* = 6; cohort 3, *N* = 9; cohort 6, *N* = 10). Each cohort was intended to receive three cycles at peptide amounts of either 300 μg (± 50), 700 μg (± 150), or 1300 μg (± 200) and administered activities of either 4.5 GBq or 6.0 GBq (± 10%) (Fig. 1; note that the DRB recommended the cohort 1 administered activity be reduced from 6.0 to 4.5 GBq due to safety concerns, and therefore the planned cohorts 2, 4, 5, 7, and 8, which would have included administered activities of more than 4.5 GBq, were not performed). Cohort 1 received three cycles at a peptide amount of 300 μg (± 50) and administered activity of 6.0 GBq (± 10%), which, as noted, was reduced to 4.5 GBq as recommended by the DRB after three patients received treatment at 6.0 GBq. Cohorts 3 and 6 investigated escalating the peptide amount and specifically whether receptor saturation occurred at given amounts: starting with 300 μg for cycle 1, then increasing to 700 μg and 1300 μg in cohorts 3 and 6, respectively, for cycle 2 before repeating the 300 μg dosing for cycle 3, all at an administered activity of 4.5 GBq. Patients in part B could receive up to two additional cycles if they showed clinical benefit and an acceptable tolerability profile without exceeding cumulative absorbed doses of 23 Gy to kidneys and 1.5 Gy to the bone marrow on central review.

In both parts, the interval between treatment cycles was 8 weeks, but could be extended to 12 weeks in case of inadequately recovered adverse events (AEs).

Up to 55 patients were to be enrolled in the trial, a minimum of six and up to 15 in part A, and a minimum of 25 and up to 40 in part B; however, these targets were not reached due to cancelled cohorts. The study was terminated in January 2022 due to the small number of ongoing patients; all patients had completed treatment and most were already transferred to the long-term, 5-year follow-up study (20 patients were enrolled in the long-term study [NCT05017662]).

Overall, three analyses were planned for the study. A first analysis was conducted with a cut-off date of 27 September 2019. A second analysis was initiated following the completion of cohorts 3 and 6 of part B of the study. A third analysis was to be performed when both parts of the study were entirely complete; however, due to cohorts 2, 4, 5, 7, and 8 not being performed, the second and third analyses were combined. The results of this combined analysis represent the complete results of both parts of this study, and are presented here. A final analysis is planned to be performed at the end of the 5-year follow-up study (estimated study completion date: April 2025).

### Study objectives

The primary objective of the study was to assess the safety and tolerability of PRRT with [^177^Lu]Lu-satoreotide tetraxetan administered in three cycles in patients with SSTR-positive NETs using standard safety and tolerability parameters: AEs according to National Cancer Institute Common Terminology Criteria for Adverse Events version 5.0 and vital signs; laboratory tests (haematology, biochemistry and urinalysis, and pituitary markers); 12-lead and Holter electrocardiogram (ECG); dose–limiting toxicities (DLTs); physical examination results; and use of concomitant medication throughout the study. A secondary objective was to undertake a preliminary assessment of the therapeutic efficacy of [^177^Lu]Lu-satoreotide tetraxetan PRRT by determination of Response Evaluation Criteria in Solid Tumours (RECIST) version 1.1 status. Secondary efficacy objectives included objective tumour response based on RECIST version 1.1 (assessed by overall response rate [ORR] and DCR) and PFS based on RECIST version 1.1. DCR was the proportion of patients with a best overall response of complete response (CR), partial response (PR), or stable disease (SD), until the end-of-core-trial/end-of-additional-cycles (EOCT/EOAC, 8 weeks after the last infusion of [^177^Lu]Lu-satoreotide tetraxetan) based on RECIST version 1.1. When SD or non-CR/non-progressive disease (PD) was believed to be the best overall response, it was assessed for a minimum of 8 weeks after initiation of [^177^Lu]Lu-satoreotide tetraxetan. Otherwise, the best overall response was considered not evaluable. ORR was the percentage of patients who had a CR or PR. PFS was calculated from the start of treatment to radiologically-confirmed progression or death.

### Study population

Eligible patients were aged ≥ 18 years with histologically confirmed, unresectable grade 1/2 gastroenteropancreatic (GEP)-NETs, typical or atypical lung carcinoids, or pheochromocytomas/paragangliomas, with documented disease progression, according to RECIST version 1.1, under prior anti-tumour therapy within the past 6 months prior to study entry. Patients should not have received further anti-tumour therapy once disease progression had been documented. Patients were required to have confirmed presence of SSTR on technically evaluable tumour lesions, documented via a positive SSTR scan. Patients were required to have at least one tumour lesion in part A and at least two tumour lesions in part B ≥ 2 cm with an uptake on SSTR imaging higher than that of normal liver parenchyma (target lesion on [^68^ Ga]Ga-DOTA-TATE or -DOTA-TOC positron emission tomography: maximum standardised uptake value [SUV_max_] ≥ 2 × the mean SUV [SUV_mean_] of liver background; or ^111^In-scintigraphy/single photon emission computed tomography: Krenning score 3 or 4 [uptake > normal liver, or uptake > spleen or kidneys, respectively [14]). Clinically, eligibility required a Karnofsky performance status of ≥ 60, a glomerular filtration rate ≥ 55 mL/min/1.73 m^2^, an estimated life expectancy of ≥ 6 months, and adequate hepatic, renal, and haematologic functions.

Key exclusion criteria were pregnancy, diagnosis of thymic NET, PRRT at any time before the study, and extensive radiotherapy or chemotherapy ≤ 3 months before study start.

### Treatment

[^177^Lu]Lu-satoreotide tetraxetan was administered on day 1 of each cycle as an intravenous infusion over 120 min. The infusion rate could be adjusted according to the investigator’s judgement in response to any acute infusion reactions. Patients were required to stop long- and short-acting somatostatin analogues at least 28 days and 24 h, respectively, prior to [^177^Lu]Lu-satoreotide tetraxetan treatment. For kidney protection, an amino acid infusion (IPN60070, also known as OPS301, containing L-arginine hydrochloride and L-lysine hydrochloride, each at a concentration of 1.25% w/v, in 2 L saline) was given over 4 h, starting 30–60 min before [^177^Lu]Lu-satoreotide tetraxetan infusion. The infusion time could be extended to 6 h at the discretion of the investigator.

The radiolabelling of [^177^Lu]Lu-satoreotide tetraxetan has been previously described [15]. [^177^Lu]Lu-satoreotide tetraxetan was initially manufactured locally on site, before later being centrally manufactured. [^177^Lu]Lu-satoreotide tetraxetan was labelled in accordance with Good Pharmacy Practice and any applicable local laws and regulations and was handled and stored at the radiopharmacy of each study centre before being handed to an investigator or a designated and suitably qualified deputy for administration.

After the first cycle, the administered activity and number of subsequent cycles of [^177^Lu]Lu-satoreotide tetraxetan were adjusted based on kidney and bone marrow dosimetry calculations; the administered activity of the next cycle could be reduced, or treatment administration could be delayed or stopped. The cumulative absorbed dose limits were 23 Gy for the kidneys and 1.5 Gy for the bone marrow.

### Study procedures and assessments

**Fig. 2:**
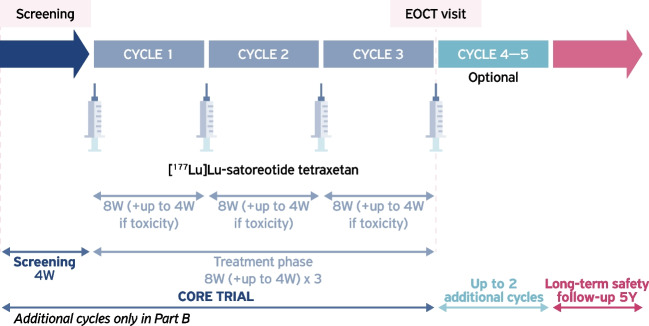
Overview of study procedures during part A and part B of the study. *EOCT* end-of-core-trial, *W* weeks, *Y* years

The study consisted of a 4-week screening period, a 6- to 10-month treatment period (plus up to an extra 6 months in part B), and an ongoing 5-year long-term follow-up (Fig. 2). All patients attended a screening visit, occurring up to 4 weeks before the first administration of [^177^Lu]Lu-satoreotide tetraxetan, in both parts. Screening assessments included review of inclusion and exclusion criteria, review of medical history, physical examination, determination of Karnofsky performance status, checks of vital signs, clinical laboratory tests, 12-lead ECG, computed tomography (CT)/magnetic resonance imaging (MRI), and a SSTR scan (unless already performed within 6 months of the first [^177^Lu]Lu-satoreotide tetraxetan administration).

After each [^177^Lu]Lu-satoreotide tetraxetan infusion, patients in both parts were monitored over 4 weeks in part A and 6 weeks in part B. For both parts, on day 1 of treatment, prior to [^177^Lu]Lu-satoreotide tetraxetan infusion, assessments included physical examination, ECG, routine clinical laboratory tests (biochemistry, haematology, pituitary marker, and urinalysis), and vital signs and AE measurements. Clinical laboratory tests, vital signs, and AE assessments were performed on days 2, 3, 4 (or 5), 7 (or 8), and 15, following [^177^Lu]Lu-satoreotide tetraxetan infusion. In part A and B, a follow-up visit was performed in week 4, in which physical examination, ECG, routine clinical laboratory tests, vital signs, and AE measurements were repeated. In part B, an additional follow-up visit was performed in week 6, in which routine clinical laboratory tests were repeated.

Eight weeks after the third treatment administration, an EOCT visit took place, which included repetition of baseline analyses. In the event that patients in part B underwent additional treatment cycles, a further assessment (EOAC visit) was performed 8 weeks after the last administration of [^177^Lu]Lu-satoreotide tetraxetan.

Radiological assessments for tumour response were performed at screening, at the end of each treatment cycle, and at the EOCT visit (or at the EOAC/early withdrawal visit), through independent central review on CT/MRI, according to RECIST version 1.1. Long-term follow-up is ongoing, in which tumour response assessments and safety evaluations will be carried out over 5 years or until disease progression or death.

Safety outcome measures included the nature, frequency, severity, and timing of treatment-emergent AEs (TEAEs), including dose-limiting AEs and AEs leading to treatment modification or withdrawal. AEs were graded using Common Terminology Criteria for Adverse Events version 5.0, and the relationship to [^177^Lu]Lu-satoreotide tetraxetan was assessed. AEs were coded using the Medical Dictionary for Regulatory Activities version 23.1. A DLT was defined as any treatment-related grade ≥ 3 AE, except for hair loss, lymphopenia, non-febrile neutropenia, and thrombocytopenia lasting < 4 weeks.

In part A, if DLTs occurred in two or fewer of the initial six patients, the remaining nine patients would continue at the administered activity. In part B, based on the first three evaluable patients in each cohort, if DLTs occurred in more than one patient, the next cohort would not be initiated. If DLTs occurred in no more than one of the patients, and two or more of the three patients had a cumulative absorbed dose in each organ-at-risk exceeding the acceptability limits (1.5 Gy in the bone marrow and 23 Gy in the kidney), patients in the next cohort would receive the same cumulative activity or less than the preceding cohort. If DLTs occurred in no more than one of the patients, and fewer than two of the three patients did not reach the cumulative absorbed dose limit in each organ-at-risk, the next cohort would be initiated as planned.

### Dosimetry

In both parts A and B of the study, patients underwent planar whole-body imaging at 4 h, and on days 1, 2, 3, and 6 after each [^177^Lu]Lu-satoreotide tetraxetan treatment cycle. Patients in part A also underwent single-photon emission computed tomography/computed tomography (SPECT/CT) at 24 h after each [^177^Lu]Lu-satoreotide tetraxetan cycle. Those in part B underwent SPECT/CT immediately before or after planar scintigraphy, at 4 h, and 1, 2, 3, and 6 days after each [^177^Lu]Lu-satoreotide tetraxetan administration. To generate pharmacokinetic data, blood samples (2 mL) were taken at 1, 5, 30, and 60 min, 4 h, and 1, 2, 3, and 6 days after each [^177^Lu]Lu-satoreotide tetraxetan administration. Time-integrated activity coefficients (TIACs) were computed by integrating the respective time-activity curves.

To assure patient safety, patient-specific dosimetry calculations were performed for organs-at-risk (kidney and bone marrow) after each treatment cycle in both parts A and B using the absorbed dose calculation features of the NUKDOS software [16]. For the bone marrow, both image-based and blood-based calculations were performed in part A [17, 18], whereas in part B, only image-based bone marrow dosimetry was performed. The image-based bone marrow absorbed dose limit of 1.5 Gy was evaluated based on the TIACs of L2-L4 (or other vertebrae in case of tumour overlay in the lumbar vertebrae region or visible bone marrow involvement). Detailed analysis of the dosimetry from this study will be reported separately.

### Statistical analyses

Statistical analysis was descriptive. It was anticipated that a total of up to 15 patients in part A and up to 40 patients in part B would be enrolled; a total of 55 patients was considered appropriate for an exploratory study, and no formal sample size calculation was performed. Safety analyses included all patients who received at least one administration of [^177^Lu]Lu-satoreotide tetraxetan. Efficacy analyses were provided in the per-protocol (PP) set (all patients who received at least one administration of [^177^Lu]Lu-satoreotide tetraxetan without any major protocol deviations). Efficacy endpoints were reported according to RECIST version 1.1 and were based on follow-up imaging assessed by independent central reviewers. Median PFS was determined in both parts and in the total study sample using Kaplan-Meier estimates. ORR and DCR and their corresponding 95% confidence intervals (CIs) were provided. Statistical analyses were performed using SAS version 9.2 or higher (SAS Institute, Cary, NC, USA). Missing values were not replaced.

## Results

### Patient characteristics

Overall, 40 patients (21 males, 19 females; median age: 62.5 years) were included in the analysis. Demographic and clinical characteristics were well balanced between patients in part A (*N* = 15) and part B (*N* = 25) (Table 1).

**Table 1:** Patient and tumour characteristics (safety analysis set)

Characteristic	Part A (*N* = 15)	Part B (*N* = 25)	Total (*N* = 40)
Age (years)			
Median (range)	65.0 (27–82)	61.0 (32–80)	62.5 (27–82)
Mean ± SD	62.7 ± 12.9	58.2 ± 13.8	59.9 ± 13.5
Sex			
Male	7 (46.7)	14 (56.0)	21 (52.5)
Female	8 (53.3)	11 (44.0)	19 (47.5)
Country			
Australia	8 (53.3)	4 (16.0)	12 (30.0)
Austria	1 (6.7)	1 (4.0)	2 (5.0)
Canada	0	1 (4.0)	1 (2.5)
Denmark	2 (13.3)	2 (8.0)	4 (10.0)
France	0	11 (44.0)	11 (27.5)
Switzerland	1 (6.7)	4 (16.0)	5 (12.5)
UK	3 (20.0)	2 (8.0)	5 (12.5)
Race			
White	15 (100.0)	24 (96.0)	39 (97.5)
Black or African American	0	1 (4.0)	1 (2.5)
Time since initial diagnosis (months)			
Median (range)	44.9 (8.1–153.9)	45.4 (5.6–157.9)	45.2 (5.6–157.9)
Mean ± SD	58.2 ± 47.8	62.8 ± 49.7	61.1 ± 48.4
Time from last relapse to screening (months)			
Median (range)	1.4 (− 0.2–3.9)	0.9 (− 0.3–5.7)	1.1 (− 0.3–5.7)
Mean ± SD	1.4 ± 1.2	1.4 ± 1.8	1.4 ± 1.6
Karnofsky performance status			
80	0	1 (4.0)	1 (2.5)
90	11 (73.3)	16 (64.0)	27 (67.5)
100	4 (26.7)	8 (32.0)	12 (30.0)
Primary tumour type			
Gastrointestinal	8 (53.3)	11 (44.0)	19 (47.5)
Pancreatic	4 (26.7)	5 (20.0)	9 (22.5)
Lung	1 (6.7)	6 (24.0)	7 (17.5)
Paraganglioma and pheochromocytoma	2 (13.3)	2 (8.0)	4 (10.0)
Unknown	0	1 (4.0)	1 (2.5)
Tumour grade			
1	4 (26.7)	5 (20.0)	9 (22.5)
2	10 (66.7)	13 (52.0)	23 (57.5)
Unknown	1 (6.7)	7 (28.0)	8 (20.0)
NET functionality			
Functioning	7 (46.7)	10 (40.0)	17 (42.5)
Non-functioning	8 (53.3)	14 (56.0)	22 (55.0)
Unknown	0	1 (4.0)	1 (2.5)
Ki-67 proliferation index (in %)			
Median (range)	4.0 (1.0–15.0)	5.0 (1.0–20.0)	5.0 (1.0–20.0)
Mean ± SD	5.5 ± 4.3	7.4 ± 5.5	6.6 ± 5.1
Not evaluable	0	4 (16.0)	4 (10.0)
Mitotic index (in mitoses per 10 HPF)			
< 2	6 (40.0)	9 (36.0)	15 (37.5)
2–20	7 (46.7)	9 (36.0)	16 (40.0)
Unknown	2 (13.3)	7 (28.0)	9 (22.5)
Prior treatments			
Somatostatin analogues	12 (80.0)	18 (72.0)	30 (75.0)
Surgery	12 (80.0)	17 (68.0)	29 (72.5)
Chemotherapy	1 (6.7)	9 (36.0)	10 (25.0)
Radiotherapy	3 (20.0)	5 (20.0)	8 (20.0)

Data are presented as *n* (%), unless otherwise specified. Percentages are calculated as *n*/*N*

*HPF* high power fields, *SD* standard deviation

### [^177^Lu]Lu-satoreotide tetraxetan treatment

**Fig. 3:**
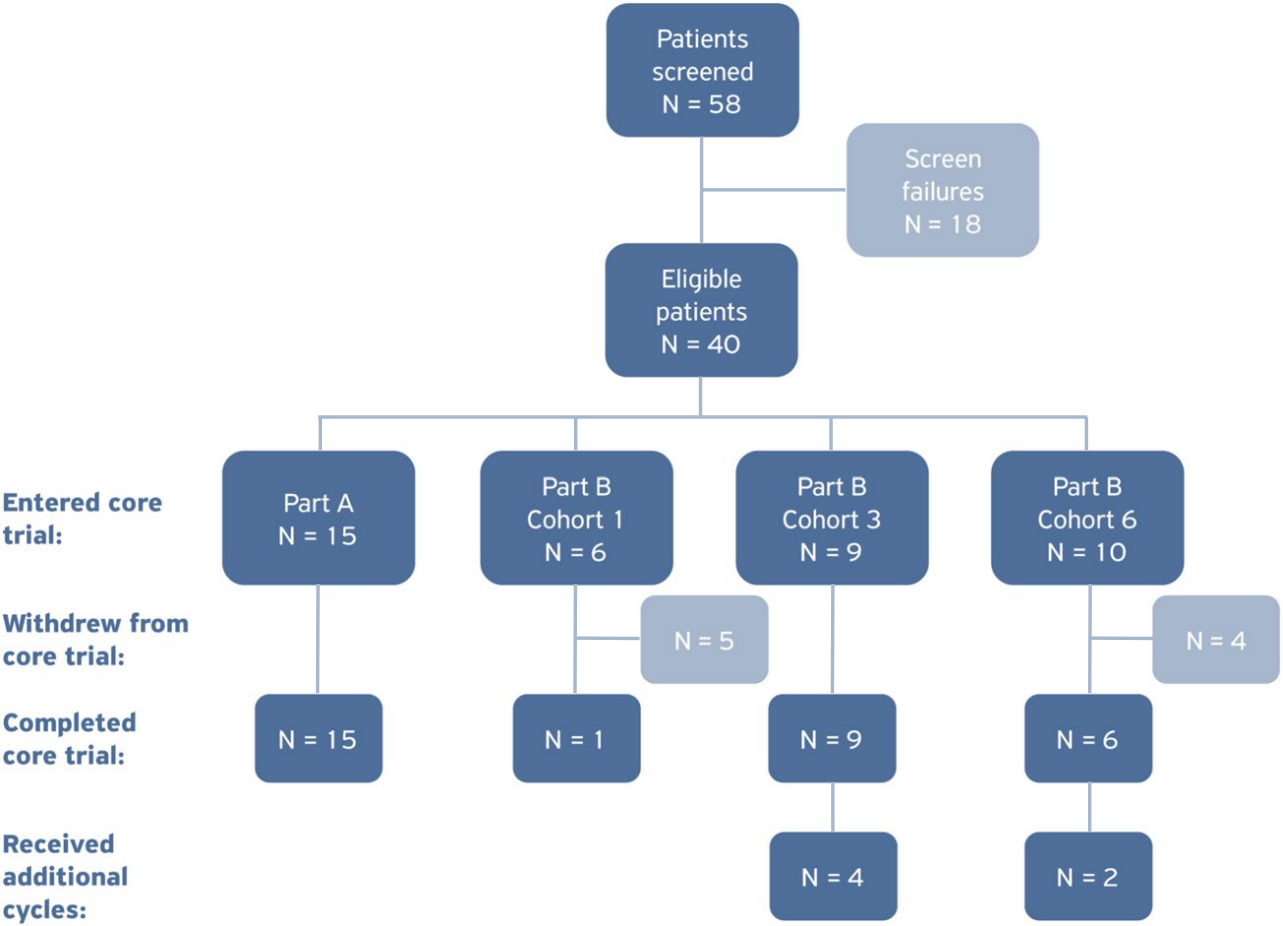
Patient disposition

The actual number of patients receiving each treatment cycle in parts A and B of the study is presented in Fig. 1. Cumulative administered activities and number of treatment cycles for each cohort are provided in Table 2. Four patients in cohort 3 received one additional treatment cycle, and two patients in cohort 6 received one and two additional treatment cycles, respectively (Table 2, Fig. 3).

**Table 2:** Overview of [^177^Lu]Lu-satoreotide tetraxetan treatment scheme (safety analysis set)

	Part A (*N* = 15)	Cohort 1 of part B (*N* = 6)	Cohort 3 of part B (*N* = 9)	Cohort 6 of part B (*N* = 10)	Part B (*N* = 25)	Total (*N* = 40)
Median cumulative administered activity (range) (GBq)	13.1 (10.3–13.5)	9.1 (4.3–15.4)	13.0 (8.5–17.6)	13.1 (4.2–20.8)	12.9 (4.2–20.8)	13.0 (4.2–20.8)
Number (%) of patients with a total number of cycles						
1 cycle	0	2 (33.3)	0	2 (20.0)	4 (16.0)	4 (10.0)
2 cycles	0	3 (50.0)	0	2 (20.0)	5 (20.0)	5 (12.5)
3 cycles	15 (100)	1 (16.7)	5 (55.6)	4 (40.0)	10 (40.0)	25 (62.5)
4 cycles	-	0	4 (44.4)	1 (10.0)	5 (20.0)	5 (12.5)
5 cycles	-	0	0	1 (10.0)	1 (4.0)	1 (2.5)

Data are presented as *n* (%), unless otherwise specified

All patients in part A completed the intended three treatment cycles (Fig. 3). In part B, nine patients discontinued treatment before completing the intended three cycles, due to severe or persistent haematotoxicity in six patients, risk of attainment of the maximal cumulative bone marrow absorbed dose of 1.5 Gy in two patients, and radiologically-confirmed PD after the second cycle in one patient. In cohort 1, the DRB recommended to reduce administered activity per cycle from 6.0 to 4.5 GBq, following the risk of the cumulative bone marrow dose exceeding 1.5 Gy after three cycles in two patients, and grade 3 thrombocytopenia in one patient following cycle 1. Patients in cohort 3 and cohort 6 received an administered activity of 4.5 GBq per cycle (Fig. 1).

### Safety

The median duration of exposure to [^177^Lu]Lu-satoreotide tetraxetan (measured as the last infusion date — first infusion date + 1 day) was 127 days (range: 1–274). All patients reported at least one TEAE (Table 3); TEAEs considered to be related to treatment occurred in 39 patients (97.5%). Overall, 17 patients (42.5%) experienced grade ≥ 3 TEAEs. No TEAEs led to treatment withdrawal during part A. During part B, seven patients (28.0%) discontinued treatment because of a TEAE (Table 3). Overall, two (5.0%) patients had TEAEs leading to reduced administered activity, five (12.5%) patients had TEAEs leading to treatment interruption, and two (5.0%) patients had TEAEs leading to treatment delay (Table 3).

**Table 3:** Overview of TEAEs and DLTs (safety analysis set)

Event	Part A (*N* = 15)	Cohort 1 of part B (*N* = 6)	Cohort 3 of part B (*N* = 9)	Cohort 6 of part B (*N* = 10)	Part B (*N* = 25)	Total (*N* = 40)
TEAEs						
Any	15 (100) [302]	6 (100) [64]	9 (100) [183]	10 (100) [147]	25 (100) [394]	40 (100) [696]
Related	15 (100) [195]	5 (83.3) [43]	9 (100) [142]	10 (100) [93]	24 (96.0) [278]	39 (97.5) [473]
TEAEs leading to drug withdrawal	0	3 (50.0) [3]	3 (33.3) [8]	1 (10.0) [1]	7 (28.0) [12]	7 (17.5) [12]
TEAEs leading to reduction of administered activity	2 (13.3) [8]	0	0	0	0	2 (5.0) [8]
TEAEs leading to treatment interruption	2 (13.3) [10]	1 (16.7) [1]	1 (11.1) [1]	1 (10.0) [2]	3 (12.0) [4]	5 (12.5) [14]
TEAEs leading to treatment delay	1 (6.7) [2]	1 (16.7) [1]	0	0	1 (4.0) [1]	2 (5.0) [3]
Grade ≥ 3 TEAEs						
Any grade 3	6 (40.0) [14]	5 (83.3) [10]	4 (44.4) [12]	2 (20.0) [2]	11 (44.0) [24]	17 (42.5) [38]
Related grade 3	5 (33.3) [8]	5 (83.3) [7]	4 (44.4) [12]	1 (10.0) [1]	10 (40.0) [20]	15 (37.5) [28]
Any grade 4	0	1 (16.7) [6]	0	1 (10.0) [1]	2 (8.0) [7]	2 (5.0) [7]
Related grade 4	0	1 (16.7) [3]	0	1 (10.0) [1]	2 (8.0) [4]	2 (5.0) [4]
Any grade 5	0	0	0	1 (10.0) [1]	1 (4.0) [1]	1 (2.5) [1]
Related grade 5	0	0	0	1 (10.0) [1]	1 (4.0) [1]	1 (2.5) [1]
Grade 3 and 4 TEAEs related to [^177^Lu]Lu-satoreotide tetraxetan^a^						
Any grade 3 and 4 related TEAEs	5 (33.3)	5 (83.3)	4 (44.4)	1 (10.0)	10 (40.0)	15 (37.5)
Blood disorders	3 (20.0)	5 (83.3)	0	1 (10.0)	6 (24.0)	9 (22.5)
Lymphopenia^b^	1 (6.7)	2 (33.3)	0	0	2 (8.0)	3 (7.5)
Thrombocytopenia^c^	1 (6.7)	2 (33.3)	0	0	2 (8.0)	3 (7.5)
Neutropenia	0	2 (33.3)	0	1 (10.0)	3 (12.0)	3 (7.5)
Anaemia	1 (6.7)	0	0	0	0	1 (2.5)
Nervous system disorders	1 (6.7)	0	0	0	0	1 (2.5)
Presyncope	1 (6.7)	0	0	0	0	1 (2.5)
Musculoskeletal disorders	0	0	1 (11.1)	0	1 (4.0)	1 (2.5)
Arthralgia	0	0	1 (11.1)	0	1 (4.0)	1 (2.5)
Back pain	0	0	1 (11.1)	0	1 (4.0)	1 (2.5)
Neoplasms benign, malignant, and unspecified	0	0	0	1 (10.0)	1 (4.0)	1 (2.5)
Acute myeloid leukaemia^d^	0	0	0	1 (10.0)	1 (4.0)	1 (2.5)
DLTs						
Any DLT	3 (20.0) [3]	0	2 (22.2) [4]	1 (10.0) [2]	3 (12.0) [6]	6 (15.0) [9]
Acute myeloid leukaemia^d^	0	0	0	1 (10.0) [2]^e^	1 (4.0) [2]	1 (2.5) [2]
Anaemia	1 (6.7) [1]^e^	0	0	0	0	1 (2.5) [1]
Arthralgia	0	0	1 (11.1) [1]	0	1 (4.0) [1]	1 (2.5) [1]
Back pain	0	0	1 (11.1) [2]	0	1 (4.0) [2]	1 (2.5) [2]
Myelodysplastic syndrome	1 (6.7) [1]^e^	0	0	0	0	1 (2.5) [1]
Platelet count decreased	0	0	1 (11.1) [1]	0	1 (4.0) [1]	1 (2.5) [1]
Presyncope	1 (6.7) [1]	0	0	0	0	1 (2.5) [1]

Data are presented as *n* (%) [number of events]. Percentages are calculated as *n*/*N*

*DLT* dose-limiting toxicity, *TACE* transcatheter arterial chemoembolisation, *TEAE* treatment-emergent adverse event

^a^Patients with more than one preferred term within a primary system organ class are only counted once for the primary system organ class

^b^Grade 3 and 4 TEAEs coded as ‘lymphocyte count decreased’ occurred in four (10.0%) patients overall

^c^Grade 3 and 4 TEAEs coded as ‘platelet count decreased’ occurred in two (5.0%) patients overall

^d^The temporal relationship between acute myeloid leukaemia and [^177^Lu]Lu-satoreotide tetraxetan seems unlikely considering the diagnosis was made 1.5 months after the first administration of [^177^Lu]Lu-satoreotide tetraxetan in a patient previously treated with myelotoxic chemotherapy (including oxaliplatin and gemcitabine) and TACE (doxorubicin)

^e^At least one of the AEs started more than 8 weeks after the last dose

Nine DLTs in six patients (15.0%) were reported across both parts. Three DLTs occurred in part A in three patients: a grade 3 presyncope recorded on the first day of [^177^Lu]Lu-satoreotide tetraxetan administration; a grade 3 anaemia reported after cycle 3, diagnosed 6.5 months after treatment initiation; and treatment-related grade 4 myelodysplastic syndrome (MDS) after cycle 3, with the time from cycle 1 initiation to diagnosis of 26.2 months.

In part B, four DLTs were reported in two patients in cohort 3. One patient had grade 3 thrombocytopenia after cycle 3, diagnosed 9 months after the start of treatment. The second patient had grade 3 lower back pain after cycle 1 and grade 3 left hip pain, and also grade 3 lower back pain after cycle 2. A patient in cohort 6 experienced a DLT that resulted in death; a 70-year-old woman with metastatic, non-functioning, grade 2 pancreatic NET who developed acute myeloid leukaemia (AML) 49 days after receiving cycle 1 of [^177^Lu]Lu-satoreotide tetraxetan given at 4.2 GBq. However, the temporal relationship between AML and [^177^Lu]Lu-satoreotide tetraxetan seems unlikely considering AML diagnosis was made approximately 1.5 months after the first [^177^Lu]Lu-satoreotide tetraxetan administration, and the patient was treated with myelotoxic chemotherapy and transcatheter arterial chemoembolisation (TACE) approximately 18 months before [^177^Lu]Lu-satoreotide tetraxetan treatment.

A 66-year-old woman in cohort 1 with functioning, grade 2 small intestinal NET, was diagnosed with B-cell lymphoblastic lymphoma 2.8 months after the completion of two cycles at a cumulative activity of 8.8 GBq (5.8 months from cycle 1 initiation to diagnosis). This event was deemed unrelated to [^177^Lu]Lu-satoreotide tetraxetan.

Overall, 46 grade ≥ 3 TEAEs occurred, of which 33 were considered to be related to [^177^Lu]Lu-satoreotide tetraxetan therapy (Table 3). The most common grade 3/4 related TEAEs were haematological events (Table 3). These haematologic AEs were transient and not associated with bleeding or infections.

There was no grade 3/4 renal toxicity observed during the study. At the end of both parts, mean change in estimated glomerular filtration rate was + 1.7 mL/min/1.73 m^2^ (range: − 16.9 to 22.0). There were no clinically meaningful changes in serum biochemistry, urinalysis, vital signs, or ECGs. No association between a higher administered peptide amount of [^177^Lu]Lu-satoreotide tetraxetan and increased or decreased toxicity was observed.

### Efficacy

**Fig. 4:**
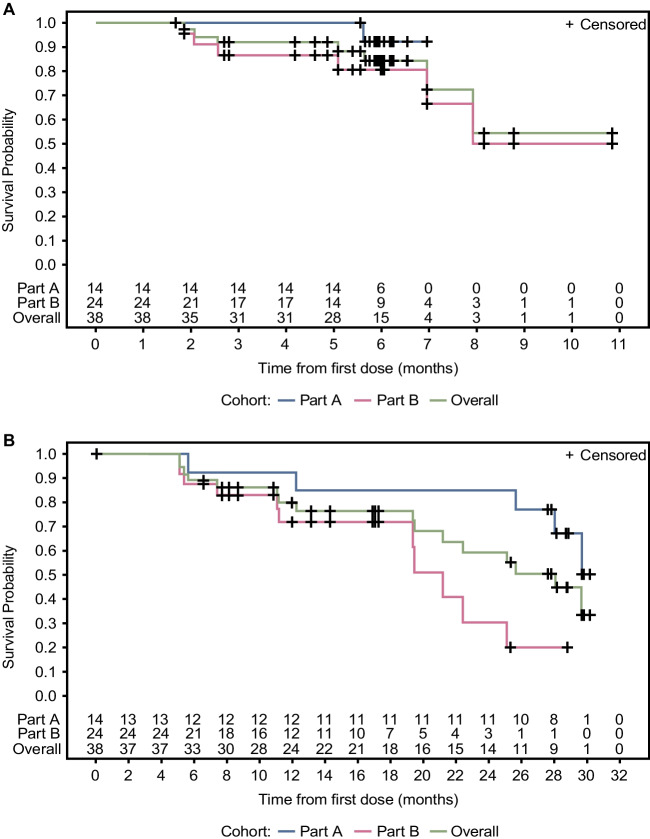
Kaplan-Meier plot for PFS based on **A** independent central review and **B** investigator assessment (RECIST version 1.1; per-protocol population). Central review was performed up until the end of treatment cycles, ending at 7 months for part A and 11 months for part B. When neither tumour progression nor death occurred, the date of the last central assessment was taken. *PFS* progression free survival, *RECIST* Response Evaluation Criteria in Solid Tumours

A total of 38 patients were included in the PP population, and could thus be evaluated for tumour response. Median PFS based on independent central review (RECIST version 1.1) was non-calculable as less than half the patients had progressed or died at the time of analysis, and central review did not continue beyond 7 months for part A and 11 months for part B, both from first dose (Fig. 4A). Median PFS based on investigator assessment (RECIST version 1.1) was 28.1 months (95% CI: 19.4–non-estimable) (Fig. 4B). At the end of treatment, for both parts A and B, no patients achieved a CR, eight a PR (21.1%), and 28 SD (73.7%), resulting in an ORR of 21.1% (95% CI: 9.6–37.3) and a DCR of 94.7% (95% CI: 82.3–99.4). Median duration of response (the time CR or PR is first observed until the time of progressed disease or death for the patients whose best overall response is CR or PR) was 17.9 months (95% CI: 6.8–non-estimable).

## Discussion

In this analysis of a phase I/II study involving 40 patients with progressive (i.e., with documented disease progression according to RECIST version 1.1, under prior anti-tumour therapy, within the past 6 months prior to study entry), previously treated, SSTR-positive NETs, [^177^Lu]Lu-satoreotide tetraxetan, administered at a median cumulative activity of 13.0 GBq over three planned cycles had a promising clinical response. Haematologic toxicity prevented increase of the administered activity in this study, with the DRB recommending to reduce the administered activity from 6.0 to 4.5 GBq in cohort 1 of part B, following the risk of the cumulative bone marrow dose exceeding 1.5 Gy after three cycles in two patients, and grade 3 thrombocytopenia in one patient following cycle 1.

[^177^Lu]Lu-DOTA-TATE’s approval for SSTR-positive GEP‑NETs in adults was based on findings from the phase III NETTER-1 trial, where it was associated with a 79% reduction in the risk for progression or death compared with high-dose octreotide long-acting release in 229 patients with well-differentiated, metastatic, midgut NETs [1, 19]. [^177^Lu]Lu-DOTA-TATE is administered at a recommended fixed activity of 7.4 GBq every 8 weeks, for four cycles [19]. However, [^177^Lu]Lu-satoreotide tetraxetan administered at 7.4 GBq was associated with prolonged grade 4 haematotoxicity in a phase I study of 20 patients with advanced, SSTR‑positive NETs, indicating that a lower activity is more appropriate for this SSTR antagonist [11]. Indeed, treatment with a reduced administered activity of [^177^Lu]Lu-satoreotide tetraxetan drastically reduced haematologic toxicity while preserving efficacy [11]. Our data have shown that 4.5 GBq is the recommended administered activity per cycle for [^177^Lu]Lu-satoreotide tetraxetan in PRRT-naïve patients with progressive NETs, since all 15 patients enrolled in part A, exposed to 4.5 GBq/300 μg of [^177^Lu]Lu-satoreotide tetraxetan for three cycles, had no treatment discontinuations, with a relatively low occurrence of severe toxicity. Our findings discourage the administration of [^177^Lu]Lu-satoreotide tetraxetan at an administered activity higher than 4.5 GBq per cycle. This follows the decision of the DRB to not continue with [^177^Lu]Lu-satoreotide tetraxetan given at 6.0 GBq per cycle, either due to the risk of bone marrow absorbed radiation doses exceeding 1.5 Gy within three cycles of treatment, or due to severe or persistent haematotoxicity. Indeed, compared to PRRT with 7.4 GBq [^177^Lu]Lu-DOTA-TATE given for four cycles, [^177^Lu]Lu-satoreotide tetraxetan administered at 4.5 GBq per cycle for three cycles appears to offer advantages, for example, in terms of reduction in radioactive nuclear waste and direct radionuclide costs, while maintaining an acceptable safety profile. A reduced number of treatment cycles also has advantages for patients, in terms of the time and cost associated with travel for each cycle of treatment, especially for those living remotely from radionuclide therapy facilities.

Here, no renal toxicity was observed, consistent with other studies of ^177^Lu-radiolabelled SSTR antagonists [10, 11, 20]. Overall, in both parts of the study, the most common grade 3/4 treatment-related AEs were haematologic (Table 3). This is consistent with the safety profile of [^177^Lu]Lu-DOTA-TATE, as lymphopenia and thrombocytopenia have been observed after [^177^Lu]Lu-DOTA-TATE therapy but do not seem to be associated with infectious complications or bleeding episodes [1, 21, 22].

Investigators identified therapy-related myeloid neoplasms (t-MN) in the form of MDS and AML in two (5.0%) patients in this trial. This finding is comparable to other studies of PRRT performed in patients with NETs [23, 24]. For instance, in a retrospective study over a 12-year period of 521 patients with metastatic neuroendocrine neoplasms who received [^90^Y]-/[^177^Lu]Lu-DOTA-TATE (median follow-up: 51 months), 25 patients (4.8%) were diagnosed with t-MN, including six cases of AML and 19 cases of MDS [23]. Prior treatment received in this retrospective study was comparable to the trial presented here; 24% received prior chemotherapy and 72% received somatostatin analogues, compared to the 25% and 75% of patients receiving prior chemotherapy and somatostatin analogues, respectively, in our study [23]. Furthermore, in another retrospective analysis of 274 patients with GEP-NETs treated with [^177^Lu]Lu-DOTA-TATE (median follow-up: 29 months), it was found that 4% of patients developed persistent haematologic dysfunction [24]. In that analysis, however, a higher proportion of patients received prior chemotherapy (90%) and radiotherapy (93%) compared with the study presented here [24]. Caution should also be taken when comparing either of these retrospective analyses to the study here, given the PRRT treatment protocols and administered activities were varied in both retrospective analyses. Prior therapies that can cause t-MN represent confounding factors in assigning causality of events.

Here, the AML diagnosis involved a patient previously treated with chemotherapy and TACE, and occurred only 1.5 months after the first [^177^Lu]Lu-satoreotide tetraxetan administration; the chemotherapy received previously by this patient included oxaliplatin (three cycles of 2 weeks duration), gemcitabine (three cycles of 2 weeks duration and three cycles of 4 weeks duration), and doxorubicin (TACE, details of cycles unknown), approximately 18 months before PRRT. This event occurred before the expected timeframe for t-MN after PRRT; two previous studies have found the median time from the first cycle of PRRT to diagnosis of t-MN to be 26 and 41 months [23, 24]. Despite the diagnosis of AML being made shortly after the first and single treatment cycle of [^177^Lu]Lu-satoreotide tetraxetan, a relationship to treatment could not be completely ruled out. Another patient was diagnosed with treatment-related grade 4 MDS after cycle 3; the time from cycle 1 initiation to diagnosis was 26.2 months, which is within a timeframe for MDS after PRRT that has been previously reported [23, 24]. The small number of patients enrolled in the present study limits estimation of the true incidence rate of t-MN in patients treated with [^177^Lu]Lu-satoreotide tetraxetan. The risk of t-MN after [^177^Lu]Lu-satoreotide tetraxetan therapy is considered small, but nevertheless warrants close patient monitoring and thoughtful benefit/risk ratio assessment. The 5-year follow-up study will provide additional data on the long-term safety of [^177^Lu]Lu-satoreotide tetraxetan.

[^177^Lu]Lu-satoreotide tetraxetan administration resulted in an excellent tumour response with a DCR of 94.7% according to RECIST version 1.1. This DCR is comparable to the DCR of 85.0% reported in the phase I study of [^177^Lu]Lu-satoreotide tetraxetan, and the DCR of 85.1% reported in a first-in-human study of another novel SSTR antagonist, [^177^Lu]Lu-DOTA-LM3, administered in 51 patients with metastatic NETs [11, 20]. In a systematic review of 15 studies evaluating the efficacy of [^177^Lu]Lu-DOTA-TATE for the treatment of inoperable or metastatic NETs, a pooled DCR of 79.1% was found [3]. These data suggest that, despite lower administered activity and fewer cycles of treatment, [^177^Lu]Lu-satoreotide tetraxetan could be at least as effective as [^177^Lu]Lu-DOTA-TATE, encouraging the pursuit of its development in progressive, SSTR-positive NETs.

The present study has limitations common to all phase I/II trials, including the small sample size, inconsistent treatment protocols, heterogeneity of the study population, and its non-randomised nature. Despite this, we show that PRRT with [^177^Lu]Lu-satoreotide tetraxetan is associated with promising clinical efficacy and a safety profile that is similar to the safety profile of [^177^Lu]Lu-DOTA-TATE, being primarily haematologically toxic.

## Conclusion

The results of this international, open-label, phase I/II study indicate that [^177^Lu]Lu-satoreotide tetraxetan administered at 4.5 GBq per cycle for a planned total of three cycles is well tolerated in PRRT-naïve patients with previously treated, progressive, SSTR-positive NETs. Haematologic toxicity was dose limiting, and it was found that administered activities above 4.5 GBq are not recommended; however, preliminary efficacy data are encouraging in suggesting that a lower number of cycles, each with a lower administered activity, of [^177^Lu]Lu-satoreotide tetraxetan may be sufficient to achieve comparable therapeutic benefit to ^177^Lu-labelled SSTR agonist PRRT. These data support further investigation of [^177^Lu]Lu-satoreotide tetraxetan and will aid the development of future trials of [^177^Lu]Lu-satoreotide tetraxetan in the treatment of advanced and/or progressive NETs.

### Supplementary Information

Below is the link to the electronic supplementary material.

Supplementary file1 (DOCX 54 KB)

## Data Availability

Qualified researchers may request access to patient-level study data that underlie the results reported in this publication. Additional relevant study documents, including the clinical study report, study protocol with any amendments, annotated case report form, statistical analysis plan, and dataset specifications may also be made available. Patient level data will be anonymized, and study documents will be redacted to protect the privacy of study participants. Where applicable, data from eligible studies are available 6 months after the studied medicine and indication have been approved in the USA and EU or after the primary manuscript describing the results has been accepted for publication, whichever is later. Further details on Ipsen’s sharing criteria, eligible studies, and process for sharing are available here (https://vivli.org/members/ourmembers/). Any request should be submitted to www.vivli.org for assessment by an independent scientific review board.
