# Supplementary material for: A phase I/II study of the safety and efficacy of [177Lu]Lu-satoreotide tetraxetan in advanced somatostatin receptor-positive neuroendocrine tumours
Source: Eur J Nucl Med Mol Imaging. 2023 Sep 18;51(1):183–95. doi: 10.1007/s00259-023-06383-1 (PMC10684626; doi:10.1007/s00259-023-06383-1)
Supplement: Supplementary file 1 — Supplementary file1 (DOCX 54 KB) [file 259_2023_6383_MOESM1_ESM.docx]

# **Original Article**

***European Journal of Nuclear Medicine and Molecular Imaging***

**Title:** A phase I/II study of the safety and efficacy of [^177^Lu]Lu-satoreotide tetraxetan in advanced somatostatin receptor-positive neuroendocrine tumours

Damian Wild,^1^ Henning Grønbæk,^2^ Shaunak Navalkissoor,^3^ Alexander Haug,^4^ Guillaume P. Nicolas,^1^ Ben Pais,^5,6*^ Catherine Ansquer,^7^ Jean-Mathieu Beauregard,^8^ Alexander McEwan,^6**^ Michael Lassmann,^9^ Daniele Pennestri,^10^ Magali Volteau,^11^ Nat P. Lenzo,^12,13^ and Rodney J. Hicks^14,15^

*^1^Division of Nuclear Medicine, ENETS Centre of Excellence, University Hospital Basel, Basel, Switzerland; ^2^Department of Hepatology & Gastroenterology, ENETS Centre of Excellence, Aarhus University Hospital, and Clinical Institute, Aarhus University, Aarhus, Denmark; ^3^Neuroendocrine Tumour Unit, ENETS Centre of Excellence, Royal Free London NHS Foundation Trust, London, United Kingdom; ^4^Department of Radiology and Nuclear Medicine, Medical University of Vienna, Vienna, Austria; ^5^SRT-Biomedical B.V., Soest, Netherlands; ^6^Ariceum Therapeutics GmbH, Berlin, Germany; ^7^CHU Nantes, Nantes Université, Médecine Nucléaire, Nantes, France​; ^8^Department of Medical Imaging, CHU de Québec – Université Laval, Quebec City, Canada; ^9^Department of Nuclear Medicine, University Hospital Würzburg, Würzburg, Germany; ^10^Ipsen, Slough, United Kingdom; ^11^Ipsen, Les Ulis, France; ^12^GenesisCare, East Fremantle, Australia; ^13^Department of Medicine, Curtin University, Perth, Australia; ^14^Department of Medicine, St Vincent’s Hospital, The University of Melbourne, and* ^15^*Department of Medicine, Central Clinical School, the Alfred Hospital, Monash University, Melbourne, Australia*

**Former affiliation: Ipsen, Hoofddorp, Netherlands; **Former affiliation: Ipsen Bioscience, Cambridge, MA, United States*

# **Supplementary Information**

**Plain Language Summary**

Neuroendocrine tumours (NETs) are uncommon cancers which develop from cells that release hormones. NETs are found in many different organs of the body. Peptide receptor radionuclide therapy (PRRT) is a treatment which delivers radiation to specific cancer cells inside the body. PRRT is designed to specifically treat tumours, such that a high dose of radiation is given to the cancer cells, while little radiation is given to the healthy surrounding cells. [^177^Lu]Lu‑satoreotide tetraxetan is a new type of PRRT which may be an effective treatment for patients with NETs.

In this clinical trial, [^177^Lu]Lu-satoreotide tetraxetan was investigated to see whether it is an effective and safe treatment for patients with NETs. Forty patients with NETs took part in the trial between March 2017 and April 2021, across eight hospitals in Australia, Austria, Canada, Denmark, France, Switzerland, and the United Kingdom. Patients were treated with different rounds of varying amounts and strength of [^177^Lu]Lu-satoreotide tetraxetan.

The results of the trial showed that [^177^Lu]Lu-satoreotide tetraxetan, when given with an appropriate amount of radioactivity, is safe for use in patients with NETs. The most common side effects of [^177^Lu]Lu-satoreotide tetraxetan were low levels of different types of blood cells. [^177^Lu]Lu‑satoreotide tetraxetan caused no serious kidney-related side effects. Additionally, [^177^Lu]Lu‑satoreotide tetraxetan was effective at treating NETs, and around 95% of patients had their disease either improve or not worsen over the course of the trial.

Overall, [^177^Lu]Lu-satoreotide tetraxetan, given with an appropriate amount of radioactivity, is a safe and potentially effective treatment for patients with NETs. These are important, encouraging findings that will help researchers to design future trials on [^177^Lu]Lu-satoreotide tetraxetan.
